# Tobacco use among people living with HIV: analysis of data from Demographic and Health Surveys from 28 low-income and middle-income countries

**DOI:** 10.1016/S2214-109X(17)30170-5

**Published:** 2017-05-08

**Authors:** Noreen D Mdege, Sarwat Shah, Olalekan A Ayo-Yusuf, James Hakim, Kamran Siddiqi

**Affiliations:** a Department of Health Sciences, Faculty of Science, University of York, Heslington, York, UK; b School of Oral Health Sciences, Sefako Makgatho Health Sciences University, Medunsa, Pretoria, South Africa; c Department of Medicine, College of Health Sciences, University of Zimbabwe, Harare, Zimbabwe

## Abstract

**Background:**

Tobacco use among people living with HIV results in excess morbidity and mortality. However, very little is known about the extent of tobacco use among people living with HIV in low-income and middle-income countries (LMICs). We assessed the prevalence of tobacco use among people living with HIV in LMICs.

**Methods:**

We used Demographic and Health Survey data collected between 2003 and 2014 from 28 LMICs where both tobacco use and HIV test data were made publicly available. We estimated the country-specific, regional, and overall prevalence of current tobacco use (smoked, smokeless, and any tobacco use) among 6729 HIV-positive men from 27 LMICs (aged 15–59 years) and 11 495 HIV-positive women from 28 LMICs (aged 15–49 years), and compared them with those in 193 763 HIV-negative men and 222 808 HIV-negative women, respectively. We estimated prevalence separately for males and females as a proportion, and the analysis accounted for sampling weights, clustering, and stratification in the sampling design. We computed pooled regional and overall prevalence estimates through meta-analysis with the application of a random-effects model. We computed country, regional, and overall relative prevalence ratios for tobacco smoking, smokeless tobacco use, and any tobacco use separately for males and females to study differences in prevalence rates between HIV-positive and HIV-negative individuals.

**Findings:**

The overall prevalence among HIV-positive men was 24·4% (95% CI 21·1–27·8) for tobacco smoking, 3·4% (1·8–5·6) for smokeless tobacco use, and 27·1% (22·8–31·7) for any tobacco use. We found a higher prevalence in HIV-positive men of any tobacco use (risk ratio [RR] 1·41 [95% CI 1·26–1·57]) and tobacco smoking (1·46 [1·30–1·65]) than in HIV-negative men (both p<0·0001). The difference in smokeless tobacco use prevalence between HIV-positive and HIV-negative men was not significant (1·26 [1·00–1·58]; p=0·050). The overall prevalence among HIV-positive women was 1·3% (95% CI 0·8–1·9) for tobacco smoking, 2·1% (1·1–3·4) for smokeless tobacco use, and 3·6% (95% CI 2·3–5·2) for any tobacco use. We found a higher prevalence in HIV-positive women of any tobacco use (RR 1·36 [95% CI 1·10–1·69]; p=0·0050), tobacco smoking (1·90 [1·38–2·62]; p<0·0001), and smokeless tobacco use (1·32 [1·03–1·69]; p=0·030) than in HIV-negative women.

**Interpretation:**

The high prevalence of tobacco use in people living with HIV in LMICs mandates targeted policy, practice, and research action to promote tobacco cessation and to improve the health outcomes in this population.

**Funding:**

South African Medical Research Council and the UK Medical Research Council.

## Introduction

The advent of and increased access to antiretroviral therapy (ART) has transformed HIV from a deadly disease to a chronic condition for many people living with HIV.^1^ With ART, people living with HIV can now have a near-normal life expectancy.^1^ However, unhealthy behaviours such as tobacco use threaten to undermine some of the gains that have been made.^2^ Smoking increases the risk of death among people living with HIV.^3^, ^4^ A study^3^ among 924 HIV-positive women on ART in the USA reported an increased risk of death due to smoking with a hazard ratio (HR) of 1·53 (95% CI 1·08–2·19). A prospective cohort^4^ of 17 995 HIV-positive individuals from Europe and North America receiving ART found a mortality rate ratio of 1·94 (95% CI 1·56–2·41) for smokers when compared with non–smokers. The average years of life lost by HIV-positive smokers compared with HIV-positive non-smokers have been estimated as 12·3 years, which is more than twice the number of years lost by HIV infection alone.^5^

People living with HIV are more susceptible to tobacco-related illnesses such as cardiovascular disease, cancer, and pulmonary disease when compared with those who are HIV-negative or with the general population.^6^, ^7^, ^8^ Furthermore, smoking among people living with HIV increases susceptibility to infections such as bacterial pneumonia, oral candidiasis, and tuberculosis.^9^, ^10^, ^11^ A case-control study^12^ among 279 ART-naive HIV-positive men in South Africa found that current smoking tripled the risk of pulmonary tuberculosis (adjusted odds ratio [OR] 3·2; 95% CI 1·3–7·9, p=0·01). Smoking also increases the risk of developing AIDS (HR 1·36; 95% CI 1·07–1·72) among people living with HIV.^3^ This increased susceptibility has been mainly attributed to biochemical mechanisms including the immunosuppressive effects of smoking and its negative impact on immune and virological response even when on ART.^13^ Behavioural mechanisms have also been suggested—for example, an association between smoking and non-adherence to antiretroviral therapy.^14^, ^15^

Few estimates are available of population-level prevalence of tobacco use among people living with HIV from low-income and middle-income countries (LMICs), where the burden of HIV and tobacco-related illnesses is greatest.^16^, ^17^, ^18^ Our study aimed to address this evidence gap for all forms of tobacco use among people living with HIV, using data from 28 nationally representative household surveys. Our study also compared tobacco use prevalence among people living with HIV to that among HIV-negative individuals.

**Research in context**

**Evidence before this study**

We searched MEDLINE (OVID platform) for articles published from inception until Dec 31, 2015, that included the terms “smoking” or “tobacco use” or “smokeless” or “cigarette” AND “HIV” or “human immunodeficiency virus” or “AIDS” or “Acquired Immune Deficiency Syndrome” in the title and were from low-income and middle-income countries (LMICs). We updated this search on Sept 30, 2016. We identified six primary research articles on the prevalence of tobacco smoking among people living with HIV that covered eight countries, of which only one article was based on national-level Demographic and Health Survey data from one country. We did not identify any multi-LMIC comparisons on the topic.

**Added value of this study**

Our study is the largest to our knowledge to report country-level and overall prevalence estimates for tobacco smoking, smokeless tobacco use, and any tobacco use among people living with HIV from 28 LMICs using nationally representative data that is comparable across countries. Our study is also the first to compare the country-level prevalence estimates for tobacco use for people living with HIV with those for HIV-negative individuals in the respective countries where such data are available.

**Implications of all the available evidence**

Findings from our study and all other identified studies confirm that for LMICs, the prevalence of tobacco use is higher among people living with HIV than among those without HIV, for both men and women. Policy, practice, and research action on tobacco cessation among people living with HIV is urgently needed to prevent the excess morbidity and mortality due to tobacco-related diseases and to improve the health outcomes in this population. This action could include exploring effective and cost-effective tobacco cessation interventions for people living with HIV that are appropriate and scalable in low-resource settings; the integration of tobacco use services within HIV programmes in LMICs including proactive identification and recording of tobacco use, as well as the provision of tobacco use cessation interventions; increasing health-care providers' awareness and competencies on provision of tobacco use cessation services among people living with HIV; increasing awareness of the harms due to tobacco use and the benefits of quitting among people living with HIV; and implementing smoke-free policies within HIV services.

## Methods

### Data sources and procedures

We did a secondary analysis of the most recent (as at December, 2015) Demographic and Health Survey (DHS) data from 28 LMICs where both tobacco use and HIV test data were made publicly available. Access to and use of this data was authorised by the DHS programme.

The DHS is designed to collect cross-sectional data that are nationally representative of the health and welfare of women of reproductive age (15–49 years), their children, and their households at about 5-year intervals across many LMICs. The DHS procedure including the two-staged sampling approach for the selection of census enumeration areas and households, questionnaire validation, data collection for household, men, and women, and data validation are comprehensively described elsewhere.^19^ In all selected households, women aged 15–49 years are eligible to participate, and those who give consent are interviewed using a women's questionnaire. In many surveys, men aged 15–54 years (or up to 59 years in some instances) from a subsample of the main survey households are also eligible to participate, and those who give consent are interviewed using a men's questionnaire. The surveys are comparable across countries through the use of standard model questionnaires and sampling methods.

In the DHS, tobacco use is ascertained by three questions to be answered “yes” or “no”. Two are about whether the respondent currently smokes cigarettes or uses any other type of tobacco. The third asks the respondent what types of tobacco they currently smoke or use, for which all tobacco types are recorded including country-specific products.^20^ HIV status data are obtained from a HIV testing protocol that undergoes a host country ethical review and provides status from informed, anonymous, and voluntary HIV testing for both women and men. Blood spots are collected on filter paper from a finger prick and transported to a laboratory for testing. The testing involves an initial ELISA test, and then retesting of all positive tests and 5–10% of the negative tests with a second ELISA. For those with discordant results on the two ELISA tests, a new ELISA or a western blot is done.

### Statistical analysis

We analysed country-level DHS data to calculate prevalence estimates for tobacco use in people living with HIV. We also computed relative prevalence ratios (as risk ratios [RRs]) comparing prevalence between HIV-positive and HIV-negative individuals.

For each country for which HIV status data could be linked to individual records in the general survey, we included data on HIV-positive and HIV-negative individuals (as confirmed by blood test results) in the analysis. We classified respondents as “tobacco smoker” if they responded “yes” to smoking cigarettes, pipes, or other country-specific smoking products such as water pipe or hookah; as “smokeless tobacco user” if their response was “yes” to the use of chew, snuff, or other country-specific smokeless tobacco products; and as “any tobacco user” if they indicated that they smoked tobacco, used smokeless tobacco, or both. These categories were not mutually exclusive, and respondents could be classified as all three.

For each country, we estimated the crude prevalence of tobacco smoking, smokeless tobacco use, and any tobacco use for males and females. We used STATA version 14 for our analyses. The analysis included sampling weights to account for differential probabilities of selection and participation, and also accounted for clustering and stratification in the sampling design.^21^ Within STATA version 14, we declared the DHS datasets as survey type from two-stage cluster sampling: the selection of census enumeration areas based on a probability (proportional to area size) and random selection of households from a complete listing of households within the selected enumeration areas.^19^ Reported estimates include the associated 95% CIs. We also computed age-standardised prevalence rates for men and women separately, using the WHO World Standard Population Distribution based on world average population between 2000 and 2025.^22^

Countries were classified geographically into the following six WHO regions: Africa, Americas, eastern Mediterranean, Europe, southeast Asia, and western Pacific. We computed pooled regional estimates for the African region only, which had a sufficient number of countries with available data for a meta-analysis. We computed these estimates in MetaXL version 5.3 by first stabilisation of the variances of the raw proportions with a double arcsine transformation and then application of a random-effects model.^23^ We assumed that the country-level estimates were different, yet related. With a random-effects meta-analysis, the SEs of the country-specific estimates are adjusted to incorporate a measure of the extent of variation among them.^24^ The amount of variation, and hence the adjustment, can be computed from the estimates and SEs from the country-level data included in the meta-analysis.^24^ We computed overall pooled prevalence estimates for all countries combined together using the same methodology as for the regional-level estimates.

To study differences in prevalence rates between HIV-positive and HIV-negative individuals, we estimated country, regional, and overall relative prevalence ratios for tobacco smoking, smokeless tobacco use, and any tobacco use separately for males and females. We used RevMan version 5.1 for this analysis using the random-effects model, as described above.

We used the *I*^2^ statistic to assess heterogeneity between country-specific estimates: values of 25% or less indicated low heterogeneity, values greater than 25% but less than 75% indicated moderate heterogeneity, and values of 75% or greater indicated high heterogeneity. We explored potential sources of heterogeneity for prevalence estimates through meta-regression analysis. This analysis tested the association between the country-level covariates, as well as year of survey (as a binary outcome: in or before 2009—which is approximately the midpoint—or after 2009), with estimated prevalence of any tobacco use. We used STATA version 14 for meta-regression.

### Role of the funding source

The funders of the study had no role in study design, data collection, data analysis, data interpretation, or writing of the report. The corresponding author had full access to all the data in the study and had final responsibility for the decision to submit for publication.

## Results

We were able to link HIV status data with the individual data in the general DHS survey for 28 LMICs, with data for men available in all LMICs apart from Cambodia. For these, our analysis provided data for 6729 HIV-positive men (aged 15–59 years) and 11 495 HIV-positive women (aged 15–49 years), as well as 193 763 HIV-negative men and 222 808 HIV-negative women for the same age groups. Of all LMICs, we were able to include 24 (52%) of 46 in Africa, two (8%) of 26 in the Americas, one (9%) of 11 in southeast Asia, and one (6%) of 18 in the western Pacific regions. Data were not available for any of the LMICs in Europe and the eastern Mediterranean. Table 1 shows the country-level characteristics of HIV-positive and HIV-negative men and women whose data were included in the analysis.

**Table 1:** General characteristics of men and women included in the analysis

	**HIV-positive**						**HIV-negative**					
	Number analysed	Mean age (years)	Urban dwellers	Within lowest household wealth quintile	Not working	No education	Number analysed	Mean age (years)	Urban dwellers	Within lowest household wealth quintile	Not working	No education
Burkina Faso, 2010	60/100	36·1/33·7	52·5%/58·7%	13·8%/10·9%	1·6%/22·5%	49·0%/61·0%	6974/8246	31·9/28·6	28·7%/26·4%	17·4%/18·3%	7·0%/25·2%	61·6%/74·1%
Burundi, 2010	56/108	40·3/33·1	34·0%/37·3%	14·2%/16·0%	2·5%/24·6%	25·7%/34·5%	4022/4401	31·2/27·7	14·5%/10·1%	16·1%/20·0%	12·7%/26·3%	31·7%/45·6%
Cambodia, 2005	../49	../32·7	../38·6%	../13·6%	../44·9%	../26·1%	../8139	../29·9	../17·6%	../17·6%	../34·6%	../19·8%
Cameroon, 2011	215/434	37·0/31·6	55·9%/61·5%	6·2%/7·5%	9·4%/28·8%	4·4%/10·5%	6730/6819	30·3/27·8	54·9%/53·2%	18·8%/16·9%	22·2%/38·7%	9·5%/21·4%
Côte d'Ivoire, 2011	127/209	42·4/32·4	56·6%/61·3%	17·1%/12·0%	7·2%/20·2%	41·0%/58·2%	4222/4446	31·1/27·9	50·9%/50·8%	19·1%/17·6%	17·5%/34·2%	35·1%/53·8%
Democratic Republic of the Congo, 2013	44/133	34·3/31·7	47·1%/53·1%	13·1%/7·3%	18·3%/33·2%	3·7%/10·8%	8278/9183	31·4/29·1	37·0%/37·3%	17·0%/18·9%	23·7%/33·0%	4·2%/15·9%
Dominican Republic, 2013	113/80	37·9/33·6	71·9%/68·5%	47·8%/33·3%	../50·2%	14·2%/17·8%	9604/8817	32·4/29·8	72·2%/75·9%	22·6%/16·0%	../52·1%	3·8%/1·9%
Ethiopia, 2011	182/358	37·2/31·7	65·7%/67·3%	3·2%/4·5%	7·8%/43·9%	22·5%/35·4%	12 816/15 147	30·4/27·6	21·5%/23·1%	16·8%/18·4%	19·8%/62·4%	32·6%/51·1%
Gabon, 2012	168/315	41·0/32·6	85·8%/89·4%	18·6%/14·8%	23·1%/38·4%	5·2%/1·3%	5331/5174	31·7/28·4	87·0%/88·3%	14·8%/14·8%	30·9%/56·6%	6·4%/5·4%
Ghana, 2003	68/120	39·3/31·7	43·5%/53·2%	13·6%/8·0%	8·1%/16·5%	13·4%/22·4%	4197/5151	30·8/29·0	44·9%/48·3%	17·5%/17·3%	25·8%/25·1%	17·4%/28·4%
Haiti, 2012	170/249	38·9/31·7	52·0%/51·1%	12·7%/9·5%	20·0%/52·9%	22·4%/15·6%	9029/9077	30·6/28·0	44·1%/47·2%	18·0%/15·6%	30·4%/59·1%	12·8%/14·8%
India, 2005–06	279/191	35·1/30·7	40·6%/43·4%	17·3%/13·8%	4·7%/41·5%	26·6%/48·8%	49 814/52 662	31·1/29·0	35·4%/32·7%	16·1%/17·0%	14·4%/62·9%	18·2%/39·7%
Kenya, 2008–09	154/318	35·2/31·4	22·1%/30·9%	7·5%/13·7%	5·4%/24·0%	3·6%/6·4%	2941/3493	29·2/28·4	25·5%/23·0%	14·6%/18·5%	13·9%/45·9%	3·8%/9·0%
Lesotho, 2009	543/997	36·2/32·0	31·7%/38·0%	12·5%/11·1%	24·4%/51·3%	17·8%/0·9%	2532/2852	27·8/26·8	27·1%/30·8%	15·4%/16·0%	39·6%/65·2%	11·9%/1·3%
Liberia, 2013	45/83	33·4/31·9	84·2%/80·6%	8·9%/7·5%	28·1%/61·1%	15·9%/44·7%	3756/4294	29·0/28·3	58·1%/59·8%	18·7%/18·0%	28·4%/44·7%	12·8%/32·9%
Malawi, 2010	530/890	36.4/32·4	32·5%/34·6%	9·1%/11·7%	7·6%/36·4%	7·7%/16·9%	5979/6506	28·1/27·3	19·9%/17·4%	14·6%/17·7%	18·3%/45·7%	6·5%/15·2%
Mali, 2012	31/66	39·1/30·1	45·0%/39·9%	15·9%/14·4%	9·4%/54·7%	64·9%/61·7%	3720/5044	33·1/28·7	24·9%/24·6%	19·6%/20·0%	10·3%/55·4%	61·4%/75·1%
Namibia, 2013	419/814	38·3/37·4	56·7%/48·8%	15·8%/22·4%	30·3%/48·6%	13·5%/8·8%	3455/4170	30·2/31·7	55·6%/54·7%	14·7%/15·5%	45·2%/60·3%	8·7%/6·5%
Niger, 2012	17/27	40·4/33·5	33·1%/57·4%	15·5%/11·7%	13·8%/80·1%	47·4%/53·2%	3509/5074	33·5/28·8	24·5%/18·6%	15·0%/17·7%	18·5%/77·9%	62·1%/80·5%
Rwanda, 2010	154/266	39·3/33·4	35·0%/35·4%	13·5%/16·0%	5·0%/29·2%	15·5%/17·3%	6142/6686	29·9/28·0	15·3%/14·4%	14·9%/18·2%	9·4%/28·4%	11·9%/15·2%
São Tomé and Príncipe, 2008–09	39/37	34·3/32·9	31·4%/27·5%	33·4%/19·4%	11·1%/46·9%	2·4%/18·1%	2121/2513	30·5/28·9	49·8%/55·1%	18·8%/17·6%	17·3%/47·1%	1·4%/5·7%
Senegal, 2010-11	26/61	39·0/35·2	40·1%/54·7%	29·3%/17·0%	16·3%/56·8%	73·7%/67·4%	4295/5529	30·0/27·9	55·3%/48·9%	15·5%/16·3%	21·1%/60·8%	38·6%/57·4%
Sierra Leone, 2013	81/141	32·8/29·0	61·9%/53·5%	9·2%/10·2%	20·3%/36·7%	40·2%/45·6%	6654/7724	31·4/28·6	36·9%/35·3%	18·7%/18·4%	20·7%/32·4%	42·6%/56·4%
Swaziland, 2006–07	704/1438	32·8/29·1	36·9%/31·3%	15·0%/16·3%	28·8%/52·6%	11·9%/10·3%	2898/3146	24·2/27·1	26·4%/24·3%	14·9%/16·0%	55·7%/64·6%	6·5%/7·2%
The Gambia, 2013	48/93	37·8/34·0	51·2%/63·1%	27·3%/21·9%	17·1%/44·1%	59·0%/57·2%	3239/4394	28·8/27·2	62·0%/55·9%	14·9%/16·6%	33·6%/55·9%	31·9%/46·4%
Togo, 2013–14	72/127	40·9/34·8	57·4%/69·1%	7·4%/2·9%	1·1%/18·6%	9·7%/28·4%	4293/4680	31·3/29·2	43·7%/44·8%	17·7%/17·6%	24·9%/29·3%	12·5%/31·2%
Zambia, 2013–14	1573/2328	36·0/32·3	61·2%/64·6%	8·3%/10·4%	15·6%/43·9%	3·3%/7·2%	12 001/13 105	29·7/27·6	44·0%/43·0%	15·9%/18·5%	27·5%/52·4%	3·6%/8·3%
Zimbabwe, 2010–11	811/1463	35·8/32·3	32·0%/34·8%	19·6%/18·2%	31·0%/55·5%	1·2%/2·0%	5234/6389	27·7/27·3	29·6%/30·7%	16·1%/18·9%	40·3%/65·4%	1·0%/2·4%

Data are presented as male/female. ..=Data not available.

Of HIV-positive men from the 27 LMICs, 27·1% (95% CI 22·8–31·7) reported any tobacco use (table 2). The crude prevalence ranged from 9·7% (Ethiopia) to 68·3% (India). The regional pooled prevalence for Africa of any tobacco use was 26·0% (22·7–29·4). Overall, 24·4% (21·1–27·8) of HIV-positive men reported smoking tobacco (table 2). We found a substantial variation in crude prevalence of current tobacco smoking across countries, from 9·7% (Ethiopia) to 54·8% (The Gambia). The regional pooled prevalence for Africa of current tobacco smoking in HIV-positive men was 24·2% (20·9–27·6). Overall, 3·4% (95% CI 1·8–5·6) of HIV-positive men reported use of smokeless tobacco (table 2). The country-level crude prevalence varied considerably and was as high as 41·4% in India. The regional pooled prevalence for Africa of current smokeless tobacco use was 2·6% (1·7–3·6).

**Table 2:** Prevalence of tobacco use in HIV-positive men by WHO region and global pooled estimate of all 27 included countries

	**Crude prevalence (95% CI)**			**Age-standardised prevalence (95% CI)**		
	Tobacco smoking	Smokeless tobacco use	Any tobacco use	Tobacco smoking	Smokeless tobacco use	Any tobacco use
**Africa**						
Burkina Faso, 2010	25·3% (16·4 to 36·9)	6·1% (1·8 to 18·9)	28·0% (18·4 to 40·2)	23·5% (15·8 to 31·1)	8·9% (4·5 to 13·2)	29·8% (21·5 to 38·1)
Burundi, 2010	10·0% (4·4 to 21·3)	10·5% (3·7 to 26·3)	18·1% (9·2 to 32·4)	12·5% (1·8 to 23·2)	9·6% (5·6 to 13·6)	19·3% (9·0 to 29·7)
Cameroon, 2011	23·4% (17·2 to 30·9)	0·7% (0·2 to 3·0)	24·1% (17·9 to 31·6)	19·2% (13·4 to 24·9)	0·6% (−0·3 to 1·5)	19·8% (14·0 to 25·6)
Côte d'Ivoire, 2011	24·9% (16·7 to 35·6)	0·5% (0·1 to 3·4)	24·9% (16·7 to 35·6)	44·3% (33·9 to 54·7)	0·2% (−0·3 to 0·7)	44·3% (33·9 to 54·7)
Democratic Republic of the Congo, 2013	24·7% (12·7 to 42·5)	3·3% (0·7 to 13·5)	28·0% (15·8 to 44·6)	17·7% (7·6 to 27·8)	1·8% (−0·7 to 4·3)	19·5% (9·6 to 29·5)
Ethiopia, 2011	9·7% (4·9 to 18·2)	0·2% (0·03 to 1·1)	9·7% (4·9 to 18·2)	8·3% (3·6 to 13·1)	0·1% (−0·04 to 0·2)	8·3% (3·6 to 13·1)
Gabon, 2012	22·0% (13·3 to 34·2)	0·0	22·0% (13·3 to 34·2)	21·1% (12·0 to 30·1)	0·0	21·1% (12·0 to 30·1)
Ghana, 2003	23·4% (14·5 to 35·4)	0·9% (0·1 to 6·4)	23·4% (14·5 to 35·4)	22·0% (12·3 to 31·7)	1·0% (−1·0 to 2·9)	22·0% (12·3 to 31·7)
Kenya, 2008–09	26·7% (17·2 to 39·0)	2·3% (0·4 to 13·6)	26·8% (17·3 to 39·1)	22·2% (14·1 to 30·3)	3·4% (−1·4 to 8·3)	22·3% (14·2 to 30·4)
Lesotho, 2009	41·1% (36·6 to 45·7)	1·0% (0·3 to 2·7)	41·1% (36·6 to 45·7)	38·8% (34·1 to 43·5)	1·1% (−0·2 to 2·5)	38·8% (34·1 to 43·5)
Liberia, 2013	23·1% (9·0 to 47·6)	0·0	23·1% (9·0 to 47·6)	23·8% (13·6 to 33·9)	0·0	23·8% (13·6 to 33·9)
Malawi, 2010	18·8% (14·9 to 23·5)	0·6% (0·2 to 1·6)	19·2% (15·2 to 23·8)	16·1% (12·2 to 20·0)	0·5% (−0·02 to 1·0)	16·4% (12·5 to 20·3)
Mali, 2012	31·6% (16·1 to 52·6)	6·7% (1·5 to 25·2)	35·3% (19·0 to 55·9)	30·3% (16·3 to 44·4)	3·9% (−1·3 to 9·1)	33·0% (18·4 to 47·7)
Namibia, 2013	20·6% (16·0 to 26·2)	2·9% (1·5 to 5·5)	23·2% (18·2 to 29·3)	20·8% (15·3 to 26·3)	3·3% (1·2 to 5·5)	24·1% (18·3 to 30·0)
Niger, 2012	33·9% (12·5 to 64·7)	2·5% (0·3 to 20·0)	36·4% (14·2 to 66·3)	21·6% (6·2 to 37·1)	1·4% (−1·9 to 4·8)	23·1% (7·5 to 38·6)
Rwanda, 2010	23·6% (17·2 to 31·5)	7·1% (3·9 to 12·7)	27·9% (21·0 to 36·0)	26·3% (17·9 to 34·6)	5·8% (2·1 to 9·4)	30·2% (21·2 to 39·2)
São Tomé and Príncipe, 2008–09	13·2% (3·2 to 40·7)	2·1% (0·3 to 15·1)	15·3% (4·5 to 41·0)	4·5% (−0·1 to 9·2)	2·3% (−2·1 to 6·7)	6·8% (0·6 to 13·1)
Senegal, 2010–11	22·0% (5·8 to 56·2)	8·0% (2·2 to 25·1)	26·5% (8·7 to 57·9)	22·6% (13·8 to 31·4)	8·5% (−0·4 to 17·5)	28·3% (17·9 to 38·7)
Sierra Leone, 2013	19·9% (12·3 to 30·7)	0·2% (0·03 to 1·8)	19·9% (12·3 to 30·7)	18·3% (10·6 to 26·0)	0·3% (−0·3 to 0·9)	18·3% (10·6 to 26·0)
Swaziland, 2006–07	26·0% (22·5 to 29·9)	5·3% (3·8 to 7·3)	30·1% (26·3 to 34·2)	21·9% (18·7 to 25·0)	4·7% (3·2 to 6·3)	25·5% (22·1 to 28·9)
The Gambia, 2013	54·8% (31·3 to 76·3)	6·0% (1·3 to 23·4)	54·8% (31·3 to 76·3)	42·1% (25·9 to 58·4)	10·8% (−0·4 to 21·9)	42·1% (25·9 to 58·4)
Togo, 2010–14	13·5% (7·2 to 23·8)	5·4% (1·7 to 16·3)	18·9% (11·2 to 30·0)	6·7% (2·9 to 10·5)	10·0% (6·2 to 13·8)	16·7% (11·9 to 21·5)
Zambia, 2013–14	24·5% (22·0 to 27·1)	1·4% (0·8 to 2·5)	25·1% (22·6 to 27·8)	22·3% (19·8 to 24·7)	1·3% (0·6 to 2·1)	22·9% (20·4 to 25·3)
Zimbabwe, 2010–11	33·1% (29·5 to 37·0)	2·9% (1·9 to 4·6)	34·8% (31·0 to 38·8)	31·5% (27·9 to 35·1)	2·8% (1·3 to 4·3)	33·0% (29·3 to 36·7)
Pooled regional estimate	24·2% (20·9 to 27·6)	2·6% (1·7 to 3·6)	26·0% (22·7 to 29·4)	..	..	..
*I*^2^ (95% CI); χ^2^ p value	86·7% (81·5 to 90·5); p<0·0001	75·6% (63·7 to 83·5); p<0·0001	86·1% (80·5 to 90·1); p<0·0001	..	..	..
**Americas**						
Dominican Republic, 2013	25·8% (16·9 to 37·4)	0·6% (0·1 to 4·3)	26·4% (17·3 to 38·1)	25·3% (16·1 to 34·5)	0·6% (−0·6 to 1·8)	25·8% (16·5 to 35·2)
Haiti, 2012	12·3% (7·4 to 19·8)	1·8% (0·7 to 4·6)	13·2% (8·1 to 20·7)	9·3% (5·2 to 13·3)	1·4% (0·1 to 2·6)	9·9% (5·7 to 14·1)
**Southeast Asia**						
India, 2005–06	40·3% (31·2 to 50·1)	41·4% (32·0 to 51·5)	68·3% (59·1 to 76·2)	37·8% (32·1 to 43·5)	37·3% (31·3 to 43·3)	60·3% (54·6 to 65·9)
**Global**						
Pooled global estimate	24·4% (21·1 to 27·8)	3·4% (1·8 to 5·6)	27·1% (22·8 to 31·7)	..	..	..
*I*^2^ (95% CI); χ^2^ p value	88·2% (84·1 to 91·3); p<0·0001	93·8% (92·0 to 95·1); p<0·0001	93·1% (91·1 to 94·7); p<0·0001	..	..	..

**Figure 1:**
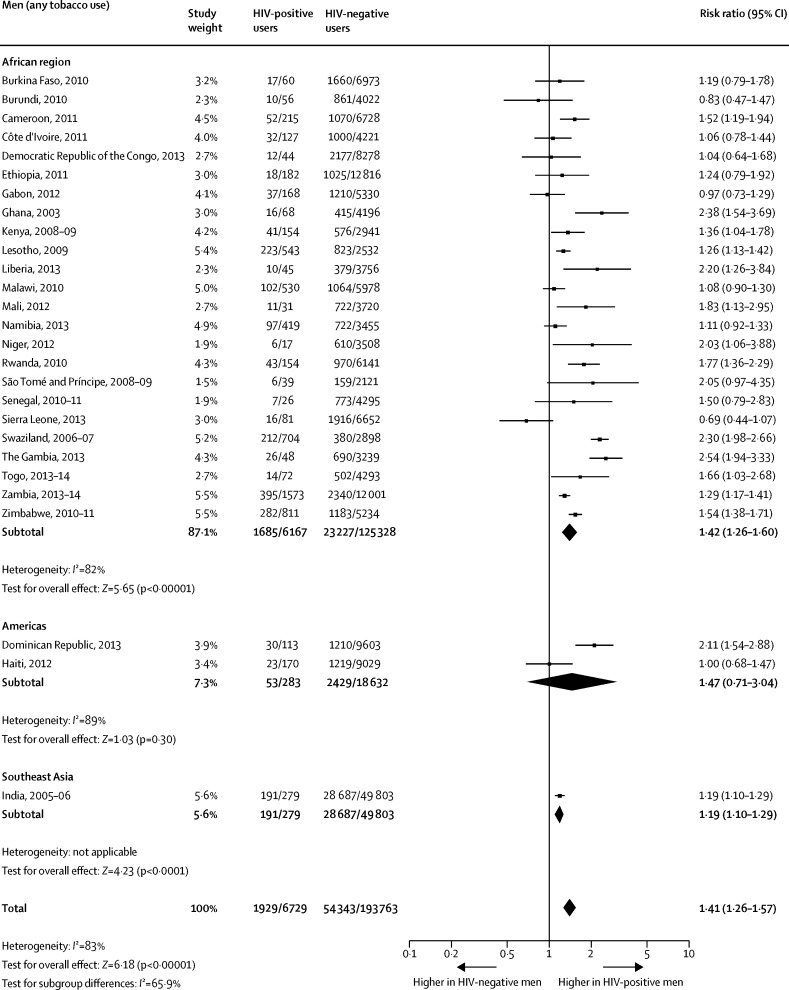
Comparison of the prevalence of any tobacco use between HIV-positive and HIV-negative men

**Figure 2:**
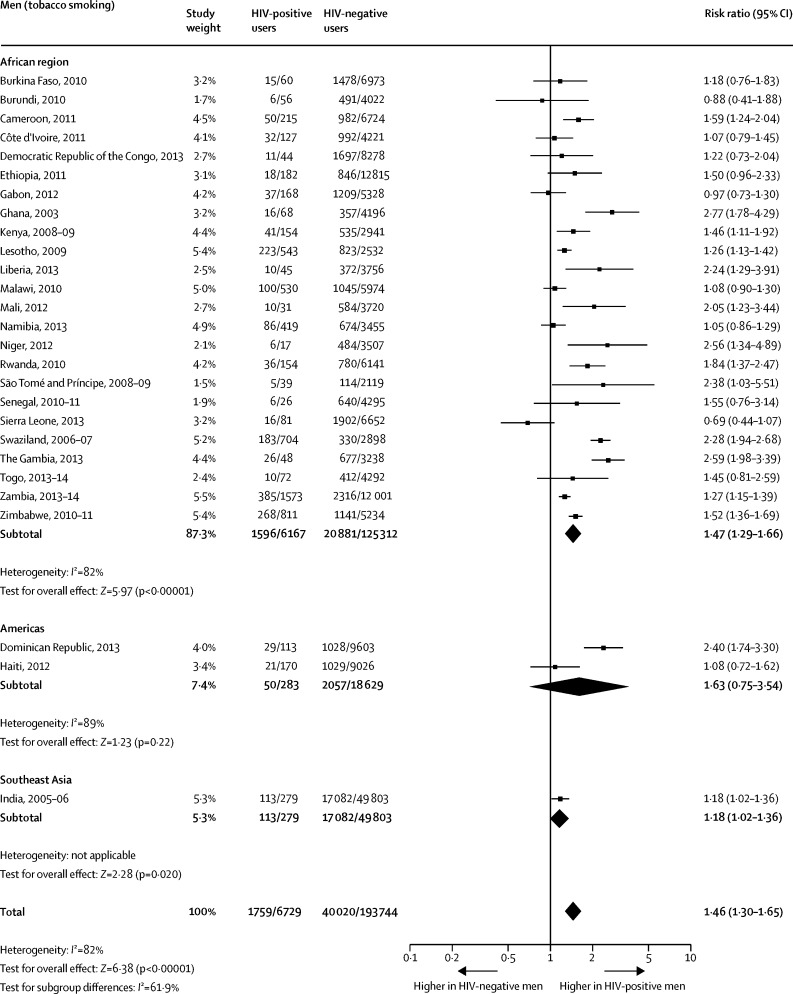
Comparison of the prevalence of tobacco smoking between HIV-positive and HIV-negative men

**Figure 3:**
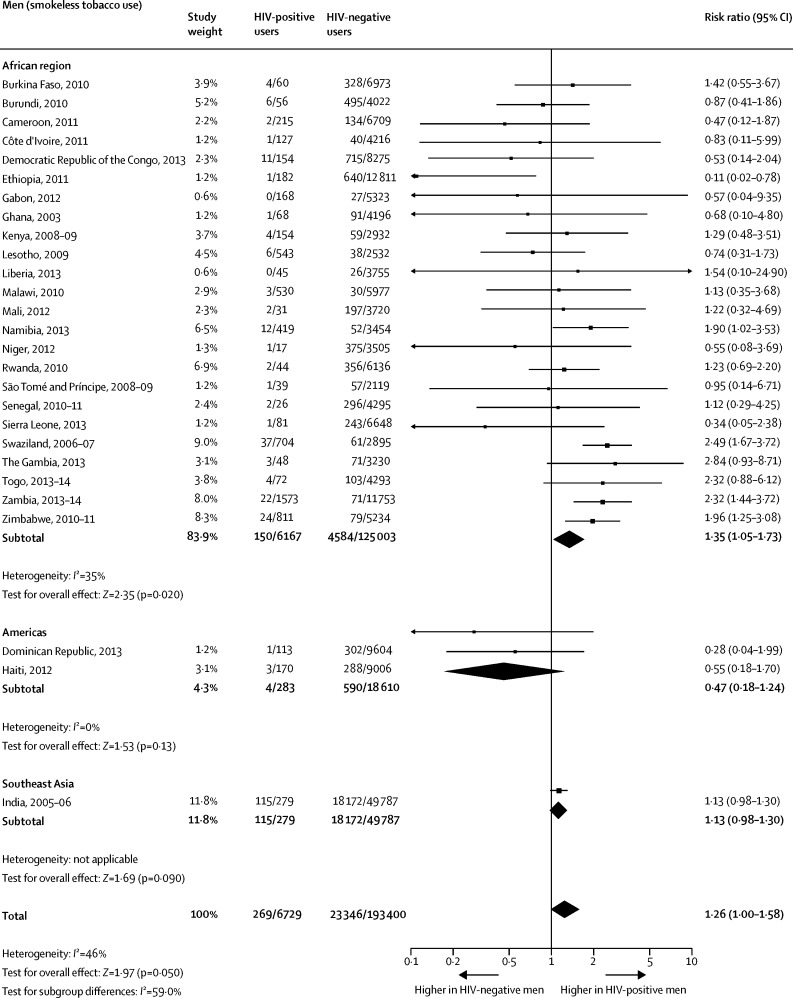
Comparison of the prevalence of smokeless tobacco use between HIV-positive and HIV-negative men

When compared with HIV-negative men, the pooled prevalence among HIV-positive men was significantly higher for any tobacco use (figure 1) and for smoking (figure 2). The difference between the two groups on smokeless tobacco use prevalence did not reach significance (figure 3). The pooled prevalences for the African region were significantly higher for HIV-positive men than for HIV-negative men for any tobacco use (figure 1), tobacco smoking (figure 2), and smokeless tobacco use (figure 3).

Of HIV-positive women from the 28 LMICs, 3·6% (95% CI 2·3–5·2) reported any tobacco use (table 3). Lesotho had the highest crude prevalence at 16·4%. The regional pooled prevalence for Africa of any tobacco use was 2·8% (1·6–4·3). Overall, 1·3% (95% CI 0·8–1·9) of HIV-positive women reported smoking tobacco (table 3). The Dominican Republic had the highest crude prevalence at 10·1%. The regional pooled prevalence for Africa of current tobacco smoking in HIV-positive women was 1·0% (0·6–1·4). Overall, 2·1% (95% CI 1·1–3·4) of HIV-positive women reported smokeless tobacco use (table 3). Lesotho had the highest crude prevalence at 15·7%. The regional pooled prevalence for Africa of current smokeless tobacco use was 1·9% (0·9–3·2).

**Table 3:** Prevalence of tobacco use in HIV-positive women by WHO region and global pooled estimate of all 28 included countries

	**Crude prevalence (95% CI)**			**Age-standardised prevalence (95% CI)**		
	Tobacco smoking	Smokeless tobacco use	Any tobacco use	Tobacco smoking	Smokeless tobacco use	Any tobacco use
**Africa**						
Burkina Faso, 2010	0·0	3·6% (1·3 to 10·2)	3·6% (1·2 to 10·2)	0·0	3·6% (0·0 to 7·2)	3·6% (0·0 to 7·2)
Burundi, 2010	0·0	8·8% (4·1 to 18·0)	8·8% (4·1 to 18·0)	0·0	6·6% (1·8 to 11·3)	6·6% (1·8 to 11·3)
Cameroon, 2011	1·0% (0·4 to 2·3)	1·7% (0·6 to 4·7)	2·7% (1·3 to 5·5)	1·0% (0·1 to 1·9)	1·7% (0·0 to 3·4)	2·7% (0·8 to 4·5)
Côte d'Ivoire, 2011	0·0	1·7% (0·5 to 6·0)	1·7% (0·5 to 6·0)	0·0	1·8% (−0·4 to 3·9)	1·8% (−0·4 to 3·9)
Democratic Republic of the Congo, 2013	0·0	1·8% (0·5 to 6·6)	1·8% (0·5 to 6·6)	0·0	1·6% (−0·3 to 3·4)	1·6% (−0·3 to 3·4)
Ethiopia, 2011	0·3% (0·1 to 0·5)	0·02% (0·00 to 0·11)	0·3% (0·1 to 0·5)	0·2% (0·1 to 0·4)	0·03% (−0·03 to 0·08)	0·2% (0·1 to 0·4)
Gabon, 2012	4·4% (1·6 to 11·3)	0·6% (0·2 to 1·4)	4·8% (1·9 to 11·4)	8·7% (4·4 to 17·0)	0·6% (0·0 to 1·2)	9·1% (0·9 to 17·3)
Ghana, 2003	0·0	0·0	0·0	0·0	0·0	0·0
Kenya, 2008–09	1·0% (0·3 to 4·1)	0·5% (0·1 to 2·1)	1·5% (0·6 to 4·3)	0·8% (−0·3 to 1·8)	0·5% (−0·2 to 1·3)	1·3% (0·0 to 2·6)
Lesotho, 2009	0·7% (0·3 to 1·5)	15·7% (13·2 to 18·7)	16·4% (13·8 to 19·4)	0·6% (0·1 to 1·1)	15·2% (12·9 to 17·5)	15·9% (13·6 to 18·1)
Liberia, 2013	0·0	0·3% (0·03 to 1·9)	0·3% (0·03 to 1·9)	0·0	0·2% (−0·2 to 0·6)	0·2% (−0·2 to 0·6)
Malawi, 2010	1·0% (0·4 to 2·6)	0·3% (0·2 to 0·8)	1·4% (0·7 to 2·9)	1·0% (0·1 to 1·9)	0·3% (0·0 to 0·6)	1·4% (0·4 to 2·3)
Mali, 2012	0·0	1·4% (0·2 to 9·8)	1·4% (0·2 to 9·8)	0·0	1·1% (−1·1 to 3·2)	1·1% (−1·1 to 3·2)
Namibia, 2013	3·2% (2·3 to 4·6)	2·1% (1·3 to 3·4)	4·7% (3·5 to 6·3)	4·1% (2·5 to 5·8)	3·3% (1·7 to 5·0)	6·2% (4·2 to 8·3)
Niger, 2012	0·0	0·0	0·0	0·0	0·0	0·0
Rwanda, 2010	1·8% (0·7 to 4·3)	3·5% (1·8 to 6·7)	5·3% (3·2 to 8·7)	1·5% (0·1 to 2·9)	2·8% (1·0 to 4·6)	4·3% (2·1 to 6·5)
São Tomé and Príncipe, 2008–09	0·7% (0·08 to 5·8)	0·0	0·7% (0·08 to 5·8)	0·6% (−0·8 to 1·9)	0·0	0·6% (−0·8 to 1·9)
Senegal, 2010–11	0·0	1·2% (0·2 to 8·8)	1·2% (0·2 to 8·8)	0·0	0·8% (−0·8 to 2·3)	0·8% (−0·8 to 2·3)
Sierra Leone, 2013	4·2% (2·0 to 8·8)	2·6% (0·7 to 9·1)	6·5% (3·2 to 12·9)	6·0% (1·2 to 10·8)	2·8% (−0·9 to 6·6)	8·6% (2·5 to 14·7)
Swaziland, 2006–07	2·2% (1·5 to 3·4)	1·5% (1·0 to 2·3)	3·6% (2·7 to 4·9)	2·0% (1·1 to 2·8)	2·2% (1·2 to 3·3)	4·1% (2·8 to 5·5)
The Gambia, 2013	0·0	0·4% (0·06 to 3·3)	0·4% (0·06 to 3·3)	0·0	0·3% (−0·4 to 1·0)	0·3% (−0·4 to 1·0)
Togo, 2013–14	0·0	1·5% (0·3 to 6·6)	1·5% (0·3 to 6·6)	0·0	1·1% (−0·5 to 2·8)	1·1% (−0·5 to 2·8)
Zambia, 2013–14	1·0% (0·6 to 1·6)	2·5% (1·8 to 3·5)	3·2% (2·4 to 4·2)	1·0% (0·5 to 1·5)	2·3% (1·6 to 3·1)	3·1% (2·3 to 4·0)
Zimbabwe, 2010–11	0·2% (0·06 to 0·5)	0·5% (0·2 to 1·1)	0·7% (0·3 to 1·2)	0·2% (0·0 to 0·3)	0·6% (0·2 to 1·0)	0·7% (0·2 to 1·1)
Pooled regional estimate	1·0% (0·6 to 1·4)	1·9% (0·9 to 3·2)	2·8% (1·6 to 4·3)	..	..	..
*I*^2^ (95% CI); χ^2^ p value	75·3% (63·3 to 83·3); p<0·0001	94·1% (92·3 to 95·4); p<0·0001	93·5% (91·5 to 95·0); p<0·0001	..	..	..
**Americas**						
Dominican Republic, 2013	10·1% (4·0 to 23·4)	..	10·1% (4·0 to 23·4)	9·1% (2·1 to 16·2)	..	9·1% (2·1 to 16·2)
Haiti, 2012	7·6% (4·3 to 13·0)	2·4% (1·1 to 5·4)	9·3% (5·7 to 14·8)	7·1% (3·2 to 11·0)	2·2% (0·4 to 4·0)	8·6% (4·4 to 12·8)
**Southeast Asia**						
India, 2005–06	2·6% (0·8 to 7·8)	9·9% (5·6 to 16·9)	12·5% (7·5 to 20·0)	2·2% (−0·2 to 4·5)	9·4% (4·4 to 14·4)	11·5% (6·2 to 16·8)
**Western Pacific**						
Cambodia, 2005	3·2% (0·7 to 13·4)	5·4% (1·3 to 19·4)	8·6% (3·1 to 22·1)	2·5% (−1·3 to 6·3)	3·5% (−0·8 to 7·7)	6·0% (0·3 to 11·7)
**Global**						
Pooled global estimate	1·3% (0·8 to 1·9)	2·1% (1·1 to 3·4)	3·6% (2·3 to 5·2)	..	..	..
*I*^2^ (95% CI); χ^2^ p value	81·3% (73·7 to 86·6); p<0·0001	93·5% (91·7 to 94·9); p<0·0001	93·3% (91·4 to 94·8); p<0·0001	..	..	..

**Figure 4:**
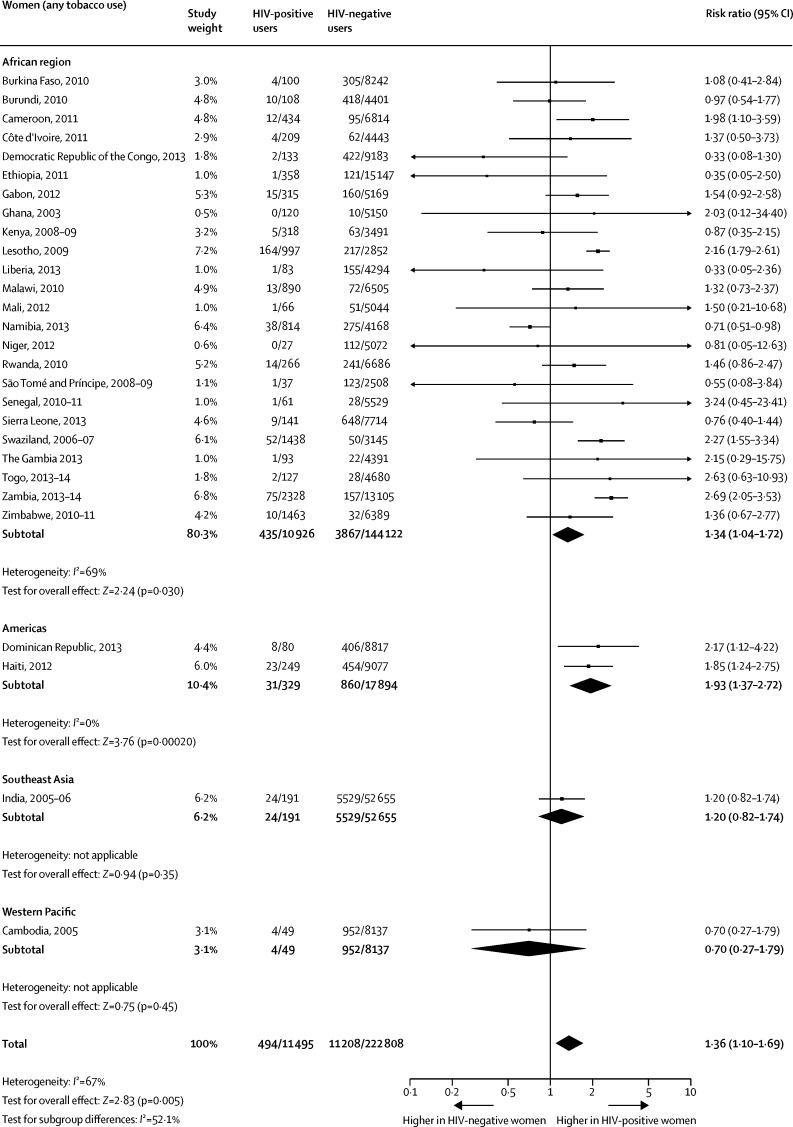
Comparison of the prevalence of any tobacco use between HIV-positive and HIV-negative women

**Figure 5:**
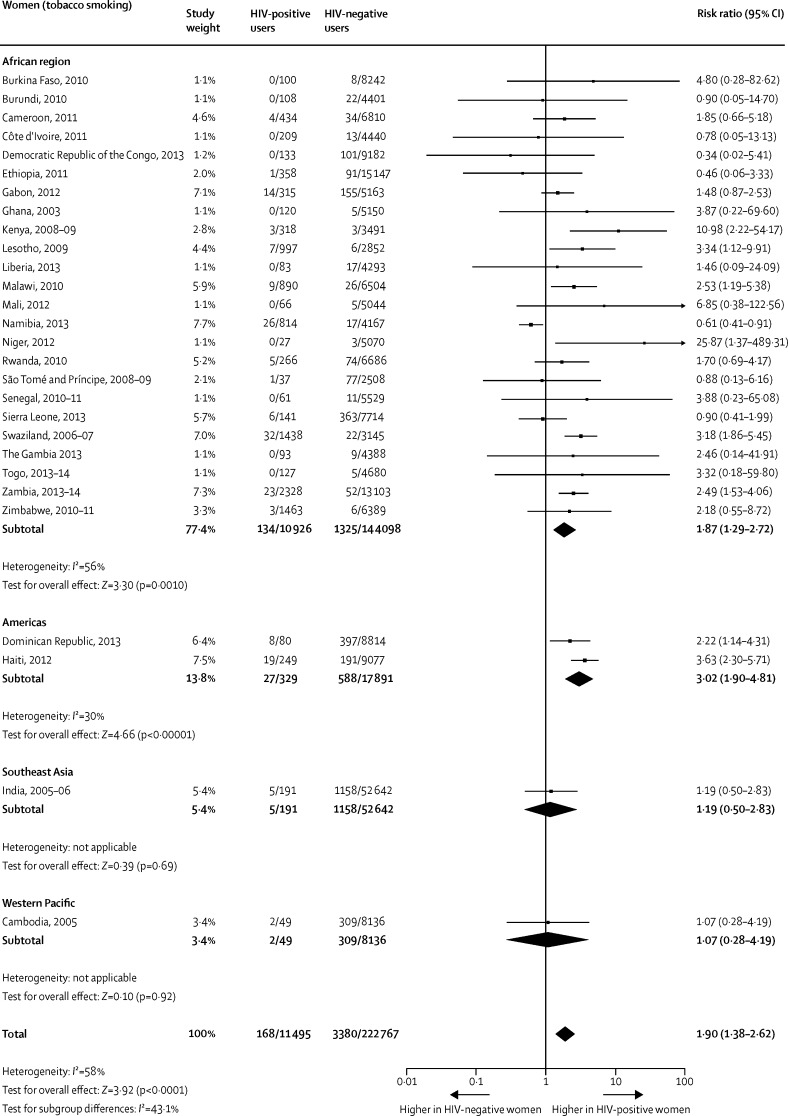
Comparison of the prevalence of tobacco smoking between HIV-positive and HIV-negative women

**Figure 6:**
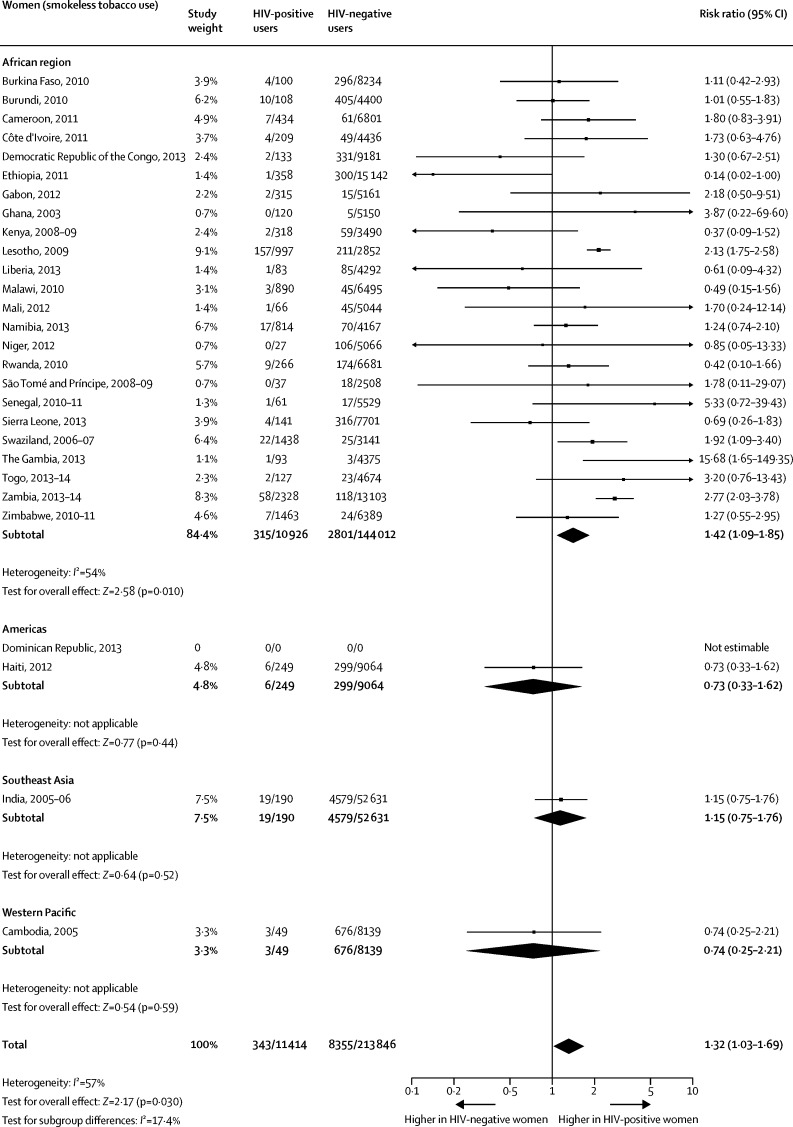
Comparison of the prevalence of smokeless tobacco use between HIV-positive and HIV-negative women

When compared with HIV-negative women, the pooled prevalences among HIV-positive women were significantly higher for any tobacco use (figure 4), current smoking (figure 5), and smokeless tobacco use (figure 6). The pooled prevalences for the African region were significantly higher for HIV-positive women than for HIV-negative women for any tobacco use (figure 4), tobacco smoking (figure 5), and smokeless tobacco use (figure 6).

We found substantial heterogeneity in prevalence estimates between countries within and across regions (table 2, table 3). However, meta-regression did not reveal any significant predictor variable for either men or women.

## Discussion

This study is the largest to our knowledge to provide up-to-date, country-specific, regional (specifically the African region) and overall prevalence estimates for tobacco smoking, smokeless tobacco use, and any tobacco use among people living with HIV using nationally representative samples from 28 LMICs. Our study shows that tobacco use prevalence in LMICs is generally higher for people living with HIV than for HIV-negative individuals, both men and women.

Other studies have reported a higher prevalence of smoking in people living with HIV than in HIV-negative individuals or in the general population. For example, in the USA, the reported prevalence of smoking among people living with HIV is 50–70% compared with about 20% in the general population or HIV-negative individuals.^3^, ^9^, ^25^, ^26^, ^27^, ^28^, ^29^, ^30^, ^31^ These differences are much higher than the differences we observed for tobacco use in our study, and are observed for both men and women. A possible explanation is that most of the developed country studies were done in non-random samples of population subgroups, such as those in a particular treatment programme or low-income groups. The differences in study results could also be attributed to differences in the profile of HIV-positive populations in developed countries compared with LMICs (eg, mostly male *vs* mostly female, respectively). Very few studies have examined tobacco use prevalence among people living with HIV in LMICs; most of these studies are also of small non-random subsamples and only present data on tobacco smoking.^32^, ^33^, ^34^, ^35^ Few of these studies make comparisons with the general population prevalence or that among HIV-negative individuals. Those studies that have used larger samples or population-level data have reported prevalence estimates that are closer to our findings. A cross-sectional survey^36^ in Zimbabwe among 6111 factory workers—88% of whom were men—found a smoking prevalence of 27% among HIV-positive individuals versus 17% among HIV-negative individuals (p<0·001). Later, Lall and colleagues^37^ reported tobacco use prevalence in India of 68% among HIV-positive men versus 58% among HIV-negative men from a secondary data analysis of 50 079 observations from the 2006 general-population National Family Health Survey data.

We found substantial variation in tobacco smoking, smokeless tobacco use, and any tobacco use prevalence between countries. Tobacco smoking was much more prevalent among men than among women—an observation that is consistent with findings from elsewhere.^38^ For women, smokeless tobacco was the primary form of tobacco use in 11 of the 28 countries. This finding has also been noted elsewhere,^21^ and potential reasons include that, in some countries, smokeless tobacco use is more socially acceptable than tobacco smoking among women.^39^ A potential for misclassification also exists, particularly owing to under-reporting in contexts in which tobacco smoking, or any tobacco use, is not socially or culturally acceptable, particularly for women.^35^, ^40^ The gender differences in product preferences need to be accounted for when designing policies and interventions for tobacco use for people living with HIV. Additionally, although the prevalence of smoking is very low overall among women living with HIV, women can be exposed to second-hand smoke, particularly if their partners smoke, and the prevalence of this exposure and associated harms needs investigation. A recent analysis^38^ of DHS data from 19 sub-Saharan Africa countries also identified other factors that increase the risk of being a smoker among people living with HIV in addition to gender, including being from poorer households and living in urban areas.

Possible reasons for high tobacco use among people living with HIV have included tobacco being used to cope with HIV-related symptoms such as neuropathic pain, as well as with anxiety, stress, and depression, all of which are higher in this population.^30^, ^41^ Studies have found that people living with HIV express an inaccurate perception of their life expectancy that affects their perceived susceptibility to the risks of tobacco use.^42^ Additionally, a study^43^ among 301 HIV-positive individuals in Mali found that, although knowledge of their HIV infection did not lead individuals to take up smoking, it negatively influenced those already smoking by increasing their consumption. These factors taken together suggest that dissemination of information on the harms of tobacco use might not be enough to reduce tobacco use in this population. The benefits of quitting also need to be emphasised either through use of targeted graphic health labels or use of culturally-appropriate mass communication media, as provided for in the guidelines for the implementation of tobacco control policies compliant with the WHO Framework Convention on Tobacco Control.

People living with HIV who use tobacco find it hard to quit and often recommence use after they have stopped.^25^, ^28^, ^31^ Pool and colleagues^44^ have published a systematic review including 14 studies (13 of which were conducted in the USA) on effectiveness of smoking cessation interventions among people living with HIV, most of which combined pharmacotherapy with nicotine replacement therapy or varenicline (or both) and psychotherapy. They found some poor-quality evidence of effectiveness in the short term but no clear evidence of effectiveness in the long term.^44^ Many interventions included in these studies were not tailored to the unique needs of people living with HIV, which was suggested as a potential reason for the poor success rates of these interventions.^16^ HIV-positive individuals face social stigma, mental and physical comorbidities, alcohol misuse, and co-dependencies on other substances, all of which influence their tobacco use behaviour, quit attempts, and successful cessation rates. Future interventions should take account of these complex social, psychological, and other health challenges faced by most people living with HIV.

In DHS data, current tobacco use status (including smoking status and smokeless tobacco use) are ascertained by self-report, which raises possibilities of under-reporting, as mentioned previously.^35^, ^40^ Smokeless tobacco was treated as a homogeneous group in our study when in fact it is a surrogate term for a diverse range of tobacco products including snuff and chewing tobacco. Additionally, countries differed in the way data on smokeless tobacco use was collected and recorded, which meant that making clear distinctions between different smokeless tobacco products proved even more difficult. Our study comprised observations from individuals who had agreed to have an HIV test as part of the DHS, and had available test results that could be linked to their tobacco use data in the general DHS dataset. This condition meant that our sample was restricted to a self-selected population and might not represent the general population—a limitation of the DHS data on HIV and not just of our analysis. Furthermore, the samples analysed for some countries were relatively small, particularly for people living with HIV. Although unlikely, a potential for selection bias still exists when individuals included in the sample were substantially different from those who were not, with respect to their smoking status. Evidence suggests that the prevalence of tobacco use could be growing in some LMICs, especially in females.^21^ Our study included data from 2003 to 2014, and therefore our prevalence estimates could be lower than the current status. Our analysis was limited to a few WHO regions, and even within these regions—with the exception of Africa—we could only include a few countries owing to unavailability of DHS data. Other countries had tobacco use data but did not have linked HIV data or did not make it publicly available, which restricted our ability to compute wider regional and global prevalence estimates.

Tobacco use leads to substantial morbidity and mortality among HIV-positive individuals.^45^ Countries with a high prevalence of tobacco use among HIV-positive populations, as highlighted by our study, should prioritise introduction of tobacco cessation in their HIV treatment plans. However, most HIV care providers are less likely to correctly identify current smokers and feel confident in their ability to influence smoking cessation than are general health workers.^46^ Given the overwhelming task of managing HIV infection and its complications, tobacco cessation might also be less of a priority from both providers' and patients' perspectives.^25^, ^42^, ^47^ Future research action to improve the health of this population could therefore include exploring effective and cost-effective tobacco cessation interventions for people living with HIV that are sustainable and scalable in low-resource settings. For policy and practice, action could include the integration of tobacco cessation within HIV programmes in LMICs including proactive identification and recording of tobacco use, as well as the provision of tobacco cessation interventions; increasing health-care providers' awareness and skills in providing cessation advice to people living with HIV; increasing awareness of the harms due to tobacco use and the benefits of quitting among people living with HIV; and implementation of smoke-free policies within HIV-treatment facilities.
